# Embelin binds to human neuroserpin and impairs its polymerisation

**DOI:** 10.1038/srep18769

**Published:** 2016-01-06

**Authors:** Giorgia Saga, Fabio Sessa, Alberto Barbiroli, Carlo Santambrogio, Rosaria Russo, Michela Sala, Samuele Raccosta, Vincenzo Martorana, Sonia Caccia, Rosina Noto, Claudia Moriconi, Elena Miranda, Rita Grandori, Mauro Manno, Martino Bolognesi, Stefano Ricagno

**Affiliations:** 1 Dipartimento di Bioscienze and CIMAINA, Università degli Studi di Milano, Milan, Italy; 2 Dipartimento di Scienze per gli Alimenti, la Nutrizione e l′Ambiente, Università degli Studi di Milano, Milan, Italy; 3 Dipartimento di Biotecnologie e Bioscienze, Università Milano-Bicocca, Milan, Italy; 4 Dipartimento di Fisiopatologia Medico-Chirurgica e dei Trapianti, Università degli Studi di Milano Italy; 5 Istituto di Biofisica, National Research Council of Italy, Palermo, Italy; 6 Dipartimento di Biotecnologie Mediche e Medicina traslazionale, Università degli Studi di Milano, Milan, Italy; 7 Dipartimento di Biologia e Biotecnologie Charles Darwin, and Istituto Pasteur – Fondazione Cenci-Bolognetti, Sapienza Università di Roma, Rome, Italy; 8 Istituto di Biofisica, National Research Council of Italy, c/o Dipartimento di Bioscienze, Università degli Studi di Milano, Milan, Italy

## Abstract

Neuroserpin (NS) is a serpin inhibitor of tissue plasminogen activator (tPA) in the brain. The polymerisation of NS pathologic mutants is responsible for a genetic dementia known as familial encephalopathy with neuroserpin inclusion bodies (FENIB). So far, a pharmacological treatment of FENIB, *i.e.* an inhibitor of NS polymerisation, remains an unmet challenge. Here, we present a biophysical characterisation of the effects caused by embelin (EMB a small natural compound) on NS conformers and NS polymerisation. EMB destabilises all known NS conformers, specifically binding to NS molecules with a 1:1 NS:EMB molar ratio without unfolding the NS fold. In particular, NS polymers disaggregate in the presence of EMB, and their formation is prevented. The NS/EMB complex does not inhibit tPA proteolytic activity. Both effects are pharmacologically relevant: firstly by inhibiting the NS polymerisation associated to FENIB, and secondly by potentially antagonizing metastatic processes facilitated by NS activity in the brain.

Neuroserpin (SERPINI1) (NS) is a member of the serpin superfamily, whose members are mainly serine-protease inhibitors^1^. Typically the native serpin fold displays a long loop named reactive center loop (RCL), which is used as a bait that is recognised and cleaved by the target protease. Such cleavage triggers the insertion of the RCL N-terminal portion into NS A β-sheet, between strands 3A and 5A (secondary structure elements are named according the accepted serpin nomenclature)^2^. Following this conformational change, the active site of the targeted protease is geometrically distorted and the acyl-enzyme adduct is not hydrolysed^3^. The resulting covalent complex is then cleared^2^.

The ability of serpins to carry out the conformational change described above, reflects the accepted notion whereby the serpin native conformation is meta-stable relative to the cleaved conformer, which is reflected by a sizeable free energy gain^4^. The meta-stability of the native conformation, which is at the root of the inhibition mechanism, is also the cause of a number of pathologies collectively known as serpinopathies^5^. In these diseases specific mutations destabilise the serpin fold and the mutated molecules tend to form polymers (Pol) and latent conformation (Lat)^6^,^7^,^8^. The latter is an uncleaved monomeric species whose uncleaved RCL is inserted into the A β-sheet, as described for the cleaved serpins^9^, in some serpins Lat plays a physiological role^10^. Conversely, the organisation of the polymeric species is not fully understood yet, and different structural models have been proposed. In all Pol models, however, the RCL is partly or completely responsible for polymerisation and is thought to be inserted into the A β-sheet of a neighbouring molecules^6^,^11^,^12^. Both Pol and Lat species are much more stable than the native conformation, and both are inactive as protease inhibitors^13^. The intracellular accumulation of mutant serpin Pol results in the formation of inclusion bodies leading to loss- and gain-of-function diseases^2^. To date no pharmacological treatment against serpin Pol formation and accumulation is available.

NS is an axonally secreted serpin, known to play a role in synaptic plasticity, memory and permeability of the neurovascular compartment^14^,^15^: it is the neuronal inhibitor of tissue plasminogen activator (tPA) in the nervous system^1^. Several mutated NS variants have been found to be responsible for an early onset dementia known as Familial Encephalopathy with Neuroserpin Inclusion Bodies (FENIB), related to NS polymer accumulation^16^. Evidences from *in vitro* and *in vivo* studies indicate that mutant NS accumulates as polymers within the endoplasmic reticulum of the expressing cells^17^,^18^,^19^,^20^. Interestingly, a new pathologic role for NS has been recently reported: in fact, the proteolysis inhibitory activity played by wild type (wt) NS was found to have a protective role for cancer cells thus promoting brain metastasis^21^.

Analogously to other serpins, native NS (Nat) displays an exposed and flexible RCL, which is inserted into β-sheet A following protease cleavage^22^. Lat and Pol species can be prepared *in vitro* by heating wt NS: such conformations display markedly higher stability than Nat^17^,^23^. Recent studies have shown that, once polymerised, NS displays an overall native-like conformation with just a moderate gain in secondary structure content, in keeping with the hypothesis that the RCL is structured in the polymers^23^,^24^. Such polymers are soluble and linear, and the kinetics of polymerisation depend both on temperature and wt NS concentration^25^. Recently Noto and co-workers reported that the NS intrinsic fluorescence can be diagnostic of the different protein conformations: in particular, the Nat to Pol conversion could be monitored by Trp/Tyr fluorescence^13^.

Several reports over the last few years focused on the effects of low molecular weight inhibitors of serpin polymerisation. Lomas and coworkers reported that a lateral hydrophobic cavity formed by strands S1A, S2A and hD is a source of instability in α_1_-antitrypsin^26^. The introduction of bulky hydrophobic residues filling this cavity resulted in stabilisation and decreased polymerisation trends. Some molecules binding that cavity selected by an *in silico* docking screen displayed an anti-polymerisation effect^27^. Recently, Andreasen and coworkers have shown that embelin (EMB), a compound originally discovered in the Japanese Ardisia herb, acts as an antagonist of PAI-1, a distinct serpin inhibitor of plasminogen activation and of tPA^28^. Upon incubation with EMB, PAI-1 loses its inhibitory activity against urokinase-type plasminogen activator (uPA) and the suicide uPA-PAI-1 covalent complex is not evidenced by SDS-PAGE analysis. The crystal structure of the complex indicates that EMB binds to PAI-1 in a pocket formed by strands 1 and 2 of the A β-sheet and by helices B and D, structural similarity and sequence identity between NS and PAI-1 are shown in figure S1.

To date no inhibitors of NS polymerisation are known thus FENIB remains a severe incurable disease. The present study thus stems from the evident need to identify a small molecule able to inhibit the polymerisation of NS pathologic mutants. Given the high level of structural homology between PAI-1 and NS, we set up to investigate the potential of EMB as an inhibitor of NS polymerisation. The data here reported indicate that the effects of EMB on NS extend well beyond what was reported for PAI-1. EMB binds to all four NS conformations (native, cleaved, latent and polymer) and in particular it affects both Pol and Lat, the NS conformers accumulated in FENIB patients. In our hands, EMB prevents the polymerisation of Nat, it dissolves preformed NS polymers and it destabilises the Lat form. The NS/EMB 1:1 stoichiometric complex tends to form small oligomers, which do not inhibit tPA activity but, conversely, are hydrolysed by tPA, an event, that may help the clearance of the NS pathologic deposits. Our results indicate that EMB exerts remarkable effects on the NS fold significantly modifying its conformational stability. Compounds displaying such effects have long been sought as starting hits to control the NS polymerisation, hence addressing therapeutic approaches for FENIB.

## Results

### Embelin affects the stability of all NS conformers

**Figure 1:**
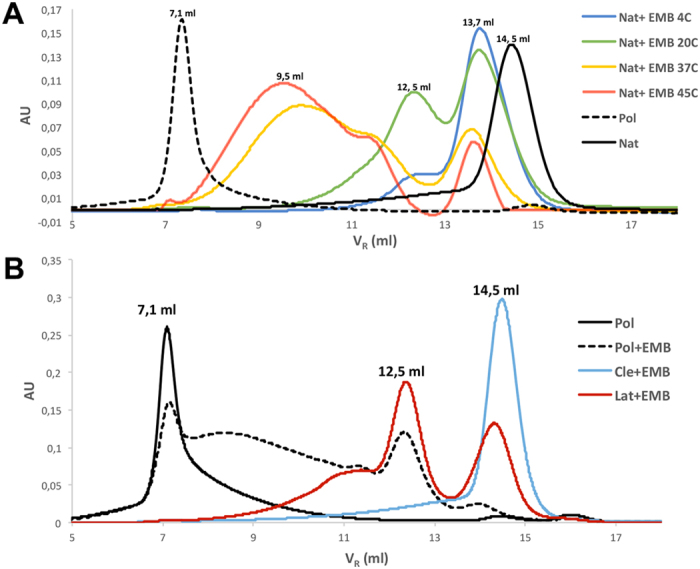
*SEC analysis of the effects of EMB on NS conformers*. (**A**) SEC profile of Nat (4 mg/ml) incubated in a saturated EMB solution overnight at different temperatures (4, 20, 37, 45, 55 °C); Nat alone and Pol are reported as controls. (**B**) SEC profile of Pol, Lat and Cle conformers (1.7 mg/ml) incubated with EMB overnight at 45 °C.

Due to the high level of structural conservation between PAI-1 and NS, we tested whether EMB exerts any structural or functional effect on wt NS. First, the typical Nat transitions to Pol and Lat conformers were explored in the presence of EMB. According to previously reported protocols, Pol and Lat conformers were concomitantly produced by heating wt NS at 45 °C and separated by SEC7^23^. The effects of EMB on Nat were monitored by size exclusion chromatography (SEC) analysis after overnight incubations at 4, 20, 37 and 45 °C in presence of 1.5 mM EMB (Fig. 1A). As standards the SEC profiles of Nat and Pol are shown: Nat alone is eluted at a volume of 14.5 ml (black line), while Pol is eluted in the column dead volume (V_R_ = 7.1 ml, dashed line), suggesting a mass over a half million Dalton in keeping with previous data^23^. After the incubation at 4 °C (blue line), NS is eluted with a major peak at 13.7 ml and a shoulder at 12.5 ml, while upon incubation at 20 °C (green line) the species at 12.5 ml became more abundant. At higher temperatures (37 and 45 °C, yellow and red lines, respectively) the height of the 13.7 ml peak decreased and a broad peak centred at 9.5 ml became the main peak. It is noteworthy that in absence of EMB, Nat is stable after incubations at low temperatures (4 and 20 °C) while yields polymeric species at higher temperatures (37–55 °C) (Fig. 1A and Fig. S2A,B). Increasing the temperature to 55 °C (Fig. S2A,B) led to SEC profiles similar to the one shown for NS incubated at 45 °C. This observation indicates that under the present conditions the oligomerisation process reaches the end state during overnight incubation at 45 °C. Interestingly, SDS-PAGE analysis of the SEC fractions indicates that the oligomers consist only of uncleaved NS (Fig. S2B). Cleaved NS (Cle), if present as in the experiment shown in figure S2B, is eluted only in the monomeric fractions, while the Lat conformer typically formed during incubation of Nat at high temperatures^23^ is virtually absent (Fig. S2B,C). Analysis of the incubation and elution data reported suggests that the oligomerisation may occur according to the following steps: first the species eluted at 13.7 ml peak is formed, followed by one eluted at 12.5 ml; finally, most NS accumulates in the broad peak centred around 9.5 ml, while the 13.7 ml peak disappears (Fig. S2C,D). Moreover, the oligomerisation rate can be accelerated by increasing Nat concentration (Fig. S2E). The data here shown indicate that the presence of EMB triggers the formation of small NS oligomers while preventing the formation of NS polymers.

In order to define the molecular mass of the species formed during the incubation with EMB, a multi-angle light scattering (MALS) analysis was performed on Nat, and on the species eluted at 13.7 and 12.5 ml (Fig. S3). MALS estimated a molecular mass of 43.1 ± 0.8 kDa for Nat and of 43.3 ± 0.9 kDa for the peak centred at 13.7 ml, both compatible with a monomeric species. The peak with a retention volume of 12.5 ml corresponds to a molecular mass of 81.5 ± 1.6 kDa, thus suggesting a dimeric species. The mass of the broad peak centred at 9.5 ml could not be estimated by MALS due to the coelution of species of higher molecular weight that, although in low amount, contribute substantially to the LS signal.

SEC analysis was also performed to evaluate the effects of EMB on the other three NS conformers. Lat, Pol and Cle were incubated at 45 °C overnight in an EMB solution. The SEC profiles shown in Fig. 1B indicate that Cle remains monomeric after the incubation (blue line), while Lat appears destabilised, since the SEC profile displays aggregates similar to those of Nat incubated with EMB (red line). Most surprisingly after overnight incubation at 45 °C the high molecular weight Pol is largely dissolved (Fig. 1B dashed line) and species comparable to those observed after the overnight incubation of Nat at 45 °C are formed. Varying the temperature of the overnight incubations affects the depolymerisation kinetics but not the final oligomeric species obtained (Fig. S2F).

### Embelin prevents NS polymerisation

**Figure 2:**
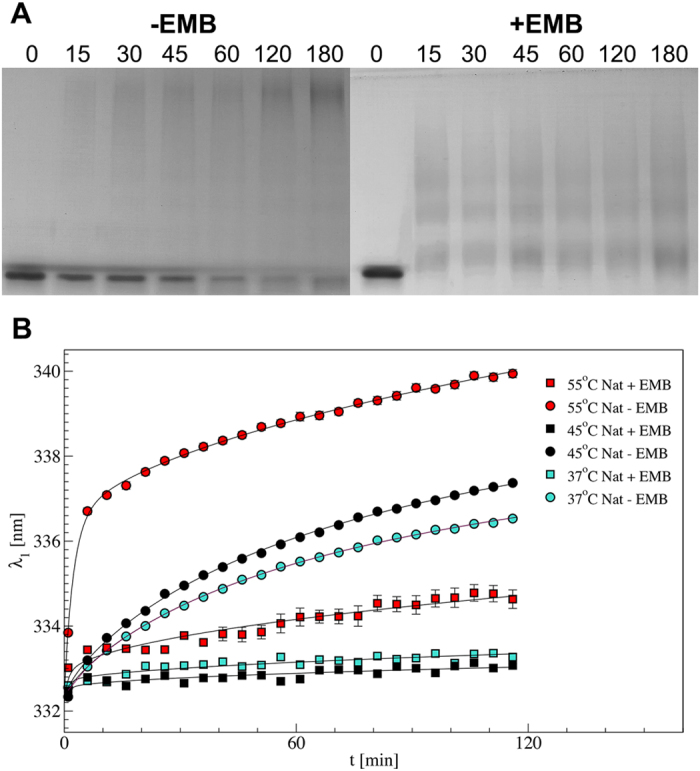
Time-course analysis of NS polymerisation. (**A**) Non-denaturing PAGE analysis of Nat polymerisation upon incubation for 0, 15, 30, 45, 60, 120 and 180 min at 45 °C in the absence (left panel) or in the presence (right panel) of saturating concentration of EMB. (**B**) Kinetics of NS polymerization monitored by PL emission (excitation wavelength 275 nm). First moment of the emission spectra of NS (5 μM, pH 8) incubated as indicated. Nat samples were either incubated directly at high temperature (circles) or previously pre-incubated with EMB at saturating concentration for 2 h followed by removal of unbound EMB by buffer exchange (squares).

The effect of EMB on the NS polymerisation kinetics was monitored by native PAGE and by intrinsic fluorescence (Fig. 2). According to previous data^17^,^23^, native PAGE monitoring the incubation of Nat at 45 °C shows polymeric species of increasing molecular weight over time, together with a small amount of monomeric Lat (Fig. 2A left panel, Fig. S2C). Instead, in the presence of EMB only small oligomers are formed starting from 15 min of incubation: their size distribution remains stable over time, with no signs of Lat formation (Fig. 2A, right panel, Fig. S2C).

We have previously reported that Trp photoluminescence (PL) emission can be used to discriminate between Nat and Pol conformers^13^, in particular a red shift of the first moment of the PL emission spectrum can be assigned to the progress of polymer formation^25^. Figure 2B shows NS polymerisation kinetics in the presence and absence of EMB. At all the tested temperatures a clear red shift in PL first moment indicates that NS polymerises during the experiment (Fig. 2B). When NS was pre-incubated with EMB (2 hours at 4 °C) such red shift was virtually absent, indicating that the Pol conformer is not formed. A measurable but much reduced inhibition of NS polymerization can be appreciated when aggregation kinetics are performed at lower EMB concentration (molar ratio 1:5 NS:EMB) (Fig. S4).

### All NS conformers interact with EMB

**Figure 3:**
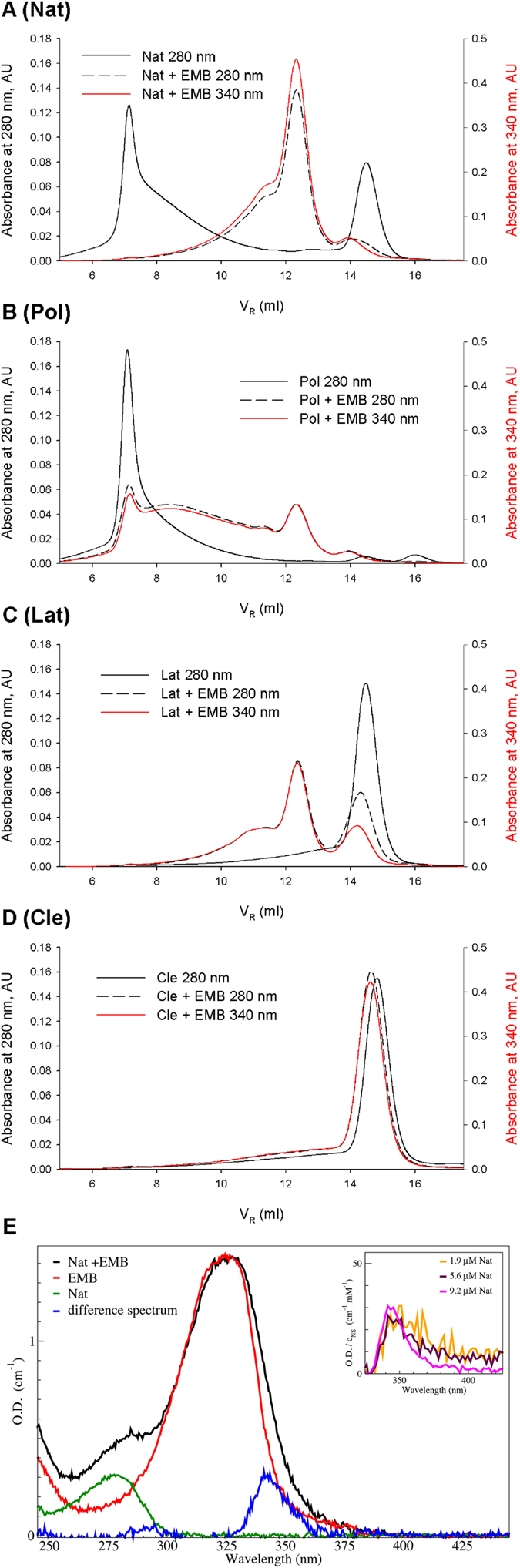
*EMB directly interacts with NS conformers*. (**A–D**) SEC profiles for identified NS conformers after overnight incubation (1.7 mg/ml) in the presence or absence of EMB at 45 °C; A: Nat, B: Pol, C: Lat, and D: Cle. The continuous black line corresponds to NS in the absence of EMB, monitored at 280 nm; the dashed black line and the red line are the SEC profiles after NS incubation with EMB, monitored at 280 nm and at 340 nm respectively. (**E**) Absorption spectra of a 8.2 μM NS solution (green line) and of a 60 μM EMB solution (red line); Nat incubated for two hours in saturated EMB solution and then washed to remove free EMB (black line); difference spectrum between the black spectrum and the previous two spectra (Nat and EMB solutions) (blue line). Inset: difference spectra calculated as above at different NS concentrations. Each spectrum is normalised for NS concentration.

In order to assess whether the effects described above are due to a direct interaction between EMB and the NS conformers or are the result of unspecific effects due to the presence of EMB, the coelution of EMB together with NS oligomers was analysed by SEC (Fig. 3). The elution of NS (with or without EMB, black continuous and dashed lines, respectively) was monitored at 280 nm, while that of EMB was followed at 340 nm (red lines). Figure 3 shows the results obtained after an overnight incubation at 45 °C for each of the four NS conformers. The profiles at 280 nm and 340 nm were perfectly superimposable, indicating that NS and EMB co-eluted hence that EMB directly bound all NS conformers. It is noteworthy that Lat is highly destabilised upon incubation with EMB, and tends to form oligomers similarly to what observed for the Nat and Pol forms. Cle is the only NS conformer, which does not form oligomers upon incubation with EMB; however a minor shift in the elution volume of Cle before and after incubation with EMB is evident (Cle panel, V_R cle_ = 14.9 ml; V_R cle+emb_ = 14.5 ml), analogously to what is observed for the Nat conformer (Fig. 1A). In summary, Fig. 3 shows that EMB is bound to each of the species that are observed after incubations: monomeric non-cleaved NS (V_R_ 13.7ml) (Fig. 3: Nat, Lat, Pol panels), monomeric Cle (Fig. 3: Cle panel) and all the oligomeric species of any size (Fig. 3: Nat, Lat and Pol panels).

Figure 3E shows the comparison of the absorption spectra of Nat and Nat incubated with EMB. The green and red spectra refer to absorption of solutions of 60 μM EMB and of 8.2 μM Nat, respectively. A Nat sample was incubated for 2 hours at 4 °C with EMB, then the excess free EMB was removed by buffer exchange; the corresponding spectrum is shown in black (Fig. 3E). Such spectrum clearly indicates that EMB directly interacts with NS. The difference spectrum of the NS/EMB complex, once the Nat and EMB individual contributions are subtracted is shown in blue. Broadening of the overall EMB absorption band, when complexed with NS, is highlighted by the appearance of a new absorption peak with a maximum at 340 nm in the difference spectrum (blue curve); such observation points to a specific interaction between NS and EMB. Moreover, the height of the 340 nm peak in the difference spectra proportionally increases with NS concentration in the sample (data not shown). Figure 3E (inset) shows that such peaks are readily superimposable if normalized for NS concentrations, thus suggesting a specific stoichiometric ratio between NS and the interacting EMB molecule(s).

### The NS/EMB complex has a defined stoichiometry

**Figure 4:**
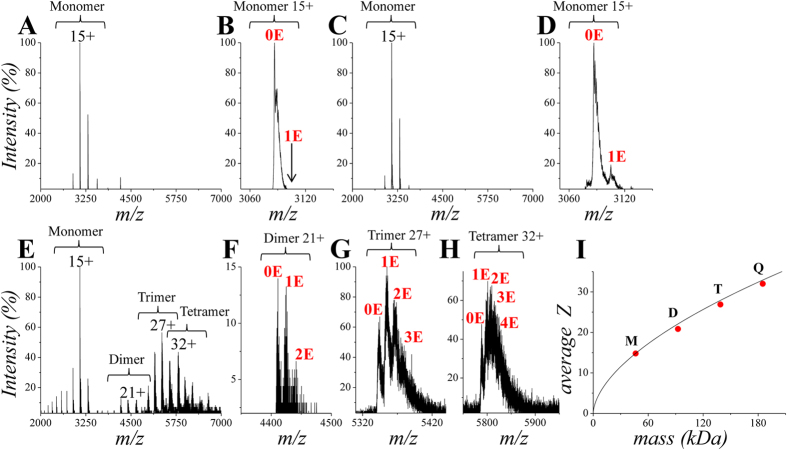
*Analysis of the NS/EMB protein-ligand complexes by native MS*. Nano-ESI-MS spectra of SEC fractions of (**A**) monomeric Nat; (**B**) enlargement of the 15+ peak shown in **(A)**, the arrow indicates the expected position of the 1:1 NS/EMB complex. (**C**) monomeric species of Nat incubated overnight with EMB (E) at 20 °C; (**D**) enlargement of the 15+ peak in **(C)**, the arrow indicates the expected position of the 1:1 NS/EMB complex; (**E**) NS oligomers after incubation as in **(C)**; (**F**) enlargement of the peak corresponding to the dimer 21+ in **(E)**; (**G**) enlargement of the peak corresponding to the trimer 27+ in **(E)**. (**H**) enlargement of the peak corresponding to the tetramer 32+ in E. (**I**) Average charge state of the monomer (M), dimer (D), trimer (T) and tetramer (Q) as a function of mass, compared to globular structures (black line^31^). Black numbers indicate charge states, red numbers indicate the molecules of bound ligand.

Mass spectrometry (MS) has been used in order to directly visualize the NS complexes with EMB and to determine its binding stoichiometry. Nat or Nat incubated overnight at 20 °C with EMB was subjected to SEC, and the monomeric and oligomeric species were analysed by electrospray-ionization mass spectrometry (ESI-MS) under non-denaturing conditions (native MS). Native MS can properly identify supramolecular complexes within heterogeneous mixtures deriving from protein-protein and protein-ligand interactions, offering combined information about the fold state and the binding properties of each detected species^29^,^30^. Figure 4A shows the spectrum of Nat (peak eluted at V_R_ 14.5 ml in SEC), corresponding to a highly pure species of 46285.5 Da, almost identical to the NS calculated mass including a N-terminal initial formylmethionine (46285 Da). The narrow charge-state distribution (CSD) is compatible with a folded protein in a compact conformation^31^. An enlargement of the main peak (15+) does not reveal any signal at the mass expected for the NS/EMB complex (Fig. 4B).

After incubation with EMB, the monomeric fraction shows a similar spectrum (Fig. 4C), but peak enlargement reveals the appearance of a new signal with an average mass shift of 292 ±1.7 Da, therefore identifiable as the 1:1 NS/EMB complex (Fig. 4D). No peaks corresponding to higher stoichiometric ratios are detectable. It is noteworthy that the acquisition of high-quality data for all the samples here reported, required the application of unusually high declustering potential (around 300 V). This peculiarity is likely due to intrinsic NS properties and not to the presence of EMB or protein aggregates. High values of declustering potential are expected to promote in-source dissociation of the protein-ligand complex during electrospray. Therefore, the relative amount of the complex is underestimated under the experimental conditions here employed. Nevertheless, these results provide a direct evidence of EMB binding to monomeric NS, and indication of complex formation with 1:1 stoichiometry. It should also be noted that the complex is detected following removal of free EMB through SEC, further suggesting that the observed complexes are stable and specific. Finally, the MS results indicate that ligand binding does not significantly affect the NS fold, since the average charge states of the free and complexed protein are closely comparable (14.8 and 15.1, respectively).

The SEC peak corresponding to higher molecular weights (V_R_ 12,5 ml), displays a drastically different spectrum relative to those described above, with peak envelopes corresponding to monomeric, dimeric, trimeric and tetrameric NS (Fig. 4E–H). The dimers, trimers and tetramers yield deconvoluted mass values of 92571.6 ±3.2 Da, 138856.3 ±2.8 Da and 185151.2 ± 14.5 Da respectively. These signals are completely absent in the spectra of the SEC peak corresponding to monomeric NS, although the final protein concentration in the eluted fractions is comparable (around 0.5 mg/ml). Thus, it can be ruled out that the observed complexes are unspecific products induced by the electrospray process. This result confirms that NS incubation in the presence of EMB mainly yields low-order protein oligomers. These oligomeric species are also complexed with EMB, as shown by the adducts in figure 4F,G. NS oligomers display a maximum of number of bound EMB molecules equal to the number of protein subunits, suggesting that 1:1 stoichiometry characterizes oligomeric as well as monomeric NS/EMB complexes. The average mass shift due to adduct formation with the ligand in the peaks of the oligomers is 294 (±2) Da per NS chain (Embelin molecular mass is 294.39 Da). The CSD also indicates that the NS molecules associated into these oligomers assemble in compact species, as the average charge states approximate the values expected for folded globular proteins of the same mass (Fig. 4I)^31^. In order to rule out unspecific interactions between NS and EMB, Nat incubated with EMB was analysed by ESI-MS without a gel filtration step, therefore maintaining the large molar excess of EMB in the sample. In this case the NS/EMB complex displaying 1:1 molar ratio was the only one found as the in gel filtered samples described above (Fig. S5). Furthermore, ESI-MS spectra of control proteins (transferrin and cytochrome C) incubated with EMB using the same incubation procedure as in Fig. 4, do not reveal any protein/EMB complex (Fig. S6).

### Embelin interaction triggers conformational changes distinct from polymer formation

**Figure 5:**
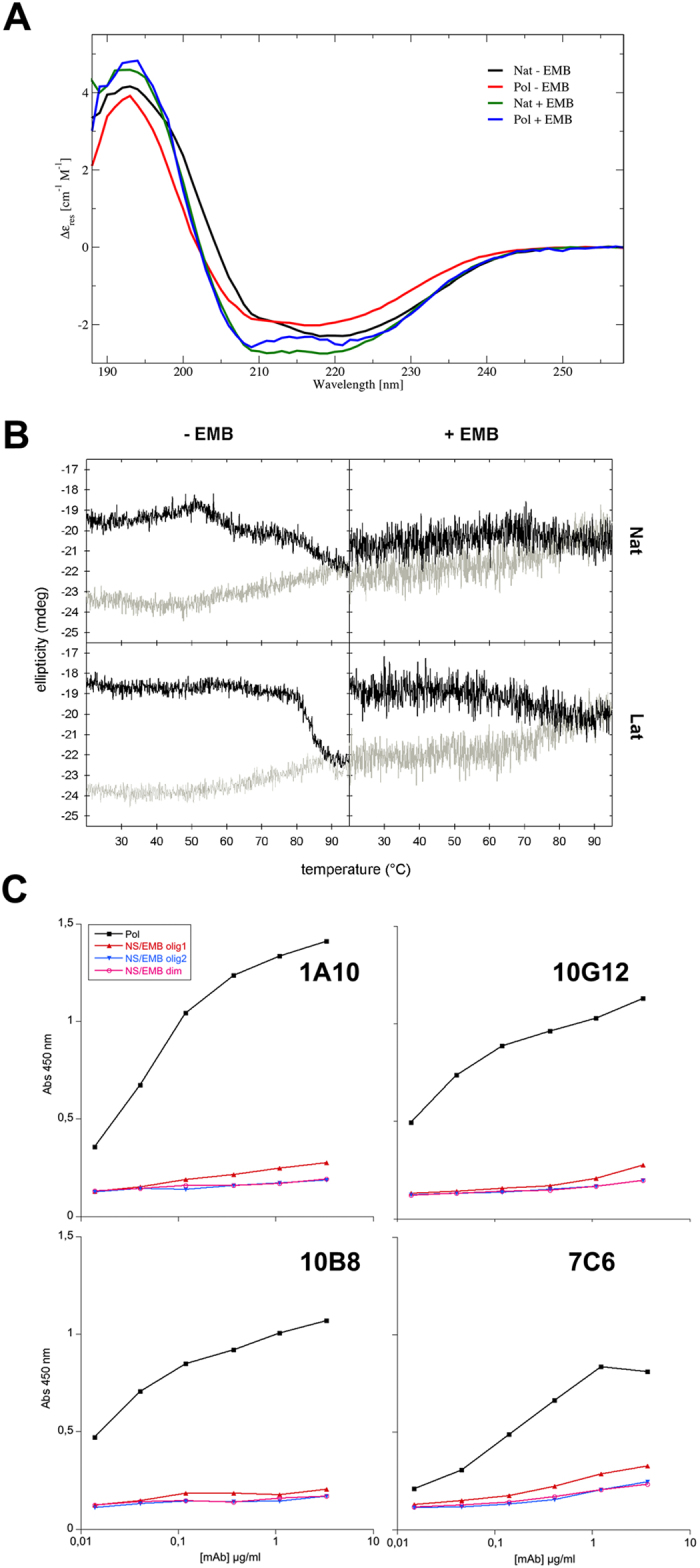
Analysis of the interaction between NS and EMB by circular dichroism (CD). (**A**) CD spectra of Nat and Pol with or without EMB: Nat (black line); Pol (red line); Nat and Pol incubated for 2 hours at 55 °C with EMB (green line and blue line, respectively). (**B**) Temperature ramps of Nat and Lat alone (−EMB) and in the presence of EMB (+EMB) monitored through far-UV CD at 218 nm. The black lines indicate the CD signal of the samples heated up from 20 to 95 °C; the grey lines correspond to samples cooled down from 95 to 20 °C. (**C**) The affinity of four different anti-NS monoclonal mAb (1A10, 10B8, 10G12 and 7C6) against NS was measured by sandwich ELISA on four gel filtered species: Pol; NS/EMB olig1 (NS/EMB largest oligomers eluted at 8.5–9 ml as in Fig. 1A); NS/EMB olig2 (NS/EMB smaller oligomers eluted at 10–10.5 ml as in Fig. 1A); and NS/EMB dim (NS/EMB dimers eluted at 12.5 ml as in Fig. 1A).

In order to investigate whether the interaction of EMB with NS triggers any conformational rearrangement, circular dichroism (CD) spectra of NS conformers in the presence or absence of EMB were recorded (Fig. 5). The far-UV CD spectra of Nat and Pol before and after incubation with EMB are shown in figure 5A. Analysis of these spectra indicates that NS does not unfold upon interaction with EMB but just minor differences at 195 and 220 nm are observable. It is noteworthy that after incubation with EMB, Nat and Pol spectra display a similar shape, suggesting that the interaction with EMB affects both Nat and Pol inducing analogous conformational rearrangements in the two conformers.

Temperature-dependent conformational changes have been monitored by CD at 218 nm for the Nat and Lat conformers, analogously to a previous report^23^. When native NS is heated up from 20 to 95 °C, the CD trace is characterized by the presence of two incremental ellipticity steps at about 55 and 85 °C (Fig. 5B top-left black line), corresponding to the Nat to Lat transition (namely, latent 45 in^23^), and to the transition from latent45 to latent85^23^. The temperature ramp of Lat (Fig. 5B bottom-left) shows only a change in the CD signal at 85 °C corresponding to the formation of latent85^23^. Such transitions are irreversible (Fig. 5B grey lines). Remarkably the temperature ramp profiles of Nat/EMB and Lat/EMB complexes behave similarly in the presence of EMB: the ellipticity changes described above are no longer visible and only a very contained increase in CD signal can be observed in the 70–80 °C range (Fig. 5B right panels). CD temperature ramps measured for Cle and Cle/EMB are identical, with no observable changes in ellipticity (data not shown).

To further compare the conformational assembly of Pol with oligomeric NS/EMB, the affinity of four different anti-NS monoclonal antibodies (mAbs)^19^ for Pol and NS/EMB oligomers was verified in a sandwich ELISA. EMB free Pol (prepared by incubation of Nat at 45 °C) was compared with dimeric and oligomeric NS/EMB (Fig. 5C). All protein samples were freshly purified by GF before being tested by ELISA. As reported in Fig. 5C, all four mAbs showed a high affinity for Pol, in agreement with previous results^19^, while none of the mAbs displayed a measurable binding to the NS/EMB species.

### The NS/EMB complex does not inhibit the activity of tPA

Next the influence of EMB on NS activity was evaluated following the inhibition of tPA hydrolytic activity. Due to direct inhibitory effect of EMB on tPA, however, free EMB could not be added to the reaction mixture (data not shown). Therefore the SEC purified monomeric NS/EMB and dimeric NS/EMB complexes-*i.e.* the species eluted at 13.5 and 12.5 ml, respectively-were tested.

**Figure 6:**
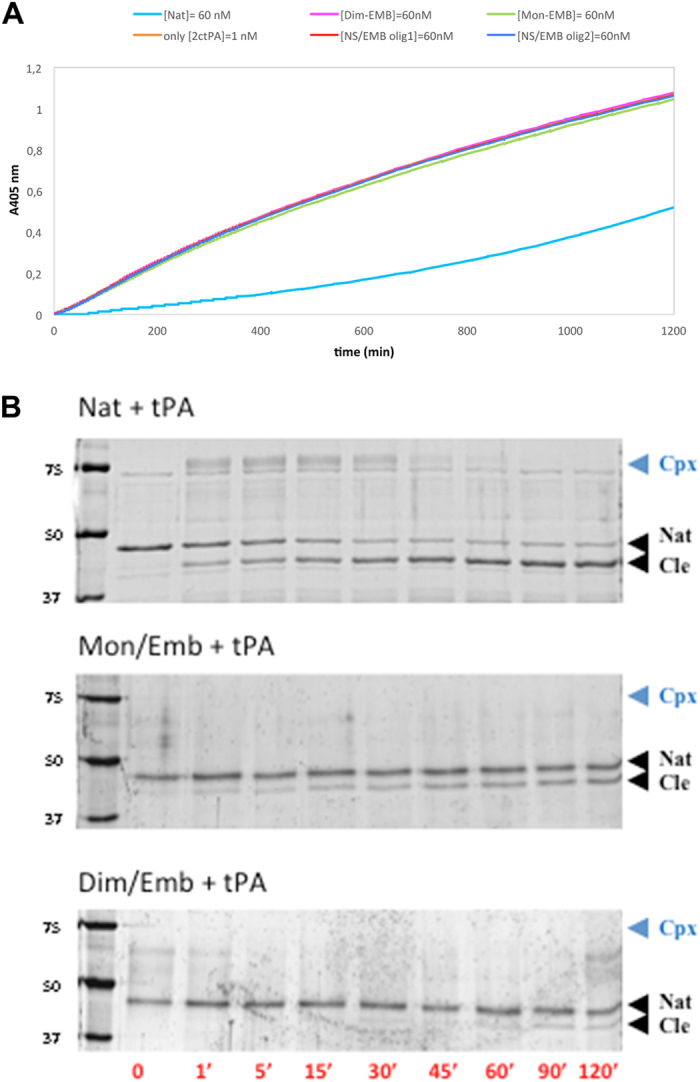
Inhibition of 2ctPA by NS/EMB complex. (**A**) Chromogenic 2ctPA inhibition assays following the hydrolysis of IPR-pNA (250 μM) by 2ctPA (1 nM) alone (orange curve) in the presence of Nat (cyan) and of NS/EMB complex at different oligomeric states (60 nM): monomer (green), dimer (magenta), largest oligomers eluted at 8.5–9 ml as in Fig. 1A (olig1, red), smaller oligomers eluted at 10–10.5 ml as in Fig. 1A (olig2, blue). (**B**) Nat, monomeric or dimeric NS/EMB complex (2.4 μM) were incubated with 2ctPA (0.4 μM) as in A and the samples analysed by SDS-PAGE. Lane 1: molecular marker; lane 2: Nat; lanes 3–10: 1, 5, 15, 30, 45, 60, 90, and 120 min time points. Nat, Cle and acyl-enzyme complex of NS with 2ctPA (cpx) bands are indicated.

First a tPA inhibitory assay was performed based on the chromogenic substrate IPR-pNA (Fig. 6A). In the presence of Nat ([NS] = 60 nM), the active form of recombinant tPA (2ctPA) is inhibited and hydrolysis of the synthetic substrate is reduced, compared to free 2ctPA, in keeping with previous results^22^ (Fig. 6A blue and orange curves, respectively). Conversely, when 2ctPA activity is assayed in the presence of monomeric, dimeric NS/EMB and oligomeric complexes (green, dashed, grey and yellow curves, respectively), the substrate is hydrolysed as efficiently as in the absence of NS (2ctPA alone, orange curve).

Formation of the covalent NS/2ctPA complex and the cleavage of NS by 2ctPA were analysed in parallel though SDS-PAGE. In the absence of EMB (Fig. 6B top panel) formation of the acyl-enzyme complex (NS/2ctPA) is evident, disappearing after one hour; after two hours Nat is almost completely cleaved (15% Nat, 85% Cle). When monomeric or dimeric NS/EMB complexes were tested, two important differences could be noticed (Fig. 6B middle and bottom panels). Firstly no covalent NS/2ctPA complex was detected from SDS-PAGE, in keeping with what has been observed for PAI-1^28^, and consistent with the lack of inhibition displayed by the NS/EMB complexes. Secondly, both monomeric and dimeric NS/EMB complexes are hydrolysed by 2ctPA, though to different extent. Within the time range of the experiment about 30% of the monomeric complex is cleaved by 2ctPA, while about 18% of the dimeric NS/EMB complex is cleaved after 120 minutes (Fig. 6B bottom panel). Thus dimeric NS/EMB is less efficiently cleaved by tPA, an indication that the RCL is less solvent exposed in the dimeric species.

## Discussion

NS polymerization is responsible for a genetic encephalopathy known as FENIB^32^. The accumulation of NS polymers leads to intracellular depositions known as Collins bodies, their size and amount correlating well with the severity of the symptoms^19^,^33^. FENIB is a progressive and fatal disease for which, to date, no treatment is available. A therapeutic intervention based on our general understanding of the molecular bases of the disease should be based on efficient inhibition of NS polymerisation. Considering the structural similarity relating the two serpins PAI-1 and NS, our work explored the inhibitory properties of EMB, a small natural molecule recently proven to interact specifically with native PAI-1^28^.

Our results show that EMB is able to bind all the NS conformers (Nat, Pol, Lat and Cle), opposite to what has been previously reported for PAI-1 where EMB binding was specific for the native conformation^28^. In the case of NS, the interaction of EMB destabilises the native conformation and trigger the formation of small oligomers. Such aggregation reaction can be accelerated by an increase in temperature or in protein concentration (Fig. 1 and S2). More specifically, we first observed the formation of a monomeric NS/EMB complex, shortly followed by the appearance of a dimeric species; at equilibrium, in all the tested temperatures and protein concentrations, an ensemble of oligomers ranging between 2 and 8 molecules was observed. All the oligomers formed upon incubation have EMB molecules bound (Fig. 3) with a 1:1 (NS:EMB) stoichiometric ratio (Fig. 4). In all oligomers the assembled NS molecules display a compact fold (Fig. 4I), with minor changes in secondary structure content (Fig. 5A). EMB binding alters the stability of the NS conformers: the conformational changes typical of NS previously reported^23^- *i.e.* secondary structure gain at 55 and 85 °C – are not observed in the presence of EMB (Fig. 5B).

The NS polymeric conformation is known to be very stable, such that chemical unfolding has been so far the only way to disaggregate NS polymers^23^. Unexpectedly, EMB triggered polymer breakdown resulting in the accumulation of smaller oligomers, which also formed upon incubation of Nat and, remarkably, Lat with EMB. Conversely Cle binds EMB but remains monomeric (Fig. 3). The inability of Cle to form oligomers may be due to the lack of an intact and uncleaved RCL, or to the very high stability of this conformer.

Several pieces of evidence suggest that the presence of EMB drives NS away from the canonical aggregation pathway resulting in polymeric and latent forms and it leads to the formation of oligomers, which are species distinct from the polymers: I) while Nat incubated at high temperatures forms increasingly bulky polymers and some Lat form, in the presence of EMB the oligomers do not grow beyond the range of 2-8 NS molecules and no Lat is observed (Fig. 2A, S1). Atomic force microscopy images show clear morphological differences between Pol and NS/EMB: the former is shaped as elongated chains the latter displays a compact ellipsoidal shape (Fig. S7). II) Pol disaggregates upon incubation with EMB (Fig. 3B and S1); and III) the difference in spectroscopic properties suggests that the NS/EMB oligomers are qualitatively distinct from NS polymers (Figs 2B, 5A). IV) Monoclonal antibodies raised against pathological S49P NS polymers that recognize polymers of NS formed by cultured cells do not bind to NS/EMB oligomers (Fig. 5C). Taken together, our results show that the interaction with EMB modifies markedly the NS conformational landscape.

Native NS conformation is known to be metastable and, in destabilised mutants (or by destabilisation of wt NS *in vitro*), it generates Pol and Lat conformers, which are known to be the most stable uncleaved species^13^,^17^,^18^. In the presence of EMB all the uncleaved conformers behave similarly and converge towards the formation of an ensemble of small oligomers (Fig. 3A–C, S1), which do not resemble in stability and behaviour the known NS polymers.

These results may bear relevant implications. It is widely accepted that incubating wt NS at temperatures ranging between 37 and 55 °C results in the formation of Lat and Pol conformers, closely resembling those produced by the pathologic mutant variants^7^,^17^,^23^. The intracellular accumulation of mutant NS polymers is toxic to neurons in the FENIB dementia, as shown in a *Drosophila* model of FENIB^19^ and suggested by the lack of an evident phenotype in NS-deficient mice^34^. The NS oligomers formed in the presence of EMB are much smaller than the polymers and highly soluble. Therefore, the NS/EMB oligomers may be more efficiently secreted and/or degraded by cells, as suggested by their susceptibility to tPA cleavage *in vitro*, instead of being further accumulated into insoluble intracellular inclusions as seen in the disease^32^,^35^,^36^. Such effects, however, require *in vivo* validation before a claim on an actual EMB detoxifying effect can be released.

Although an ideal intervention for FENIB should prevent polymer formation without affecting the physiological activity of monomeric NS, the observed lack of NS inhibitory activity on tPA in the presence of EMB might underlie an unexpected therapeutic application. Brain metastasis is one of the most devastating complications of primary cancer^37^. A recent report uncovered a role for NS in facilitating the propagation of brain metastases through the inhibition of tPA proteolytic activity^21^; in this context, NS removal reduced drastically the probability of brain metastasis. Thus, we propose that a low molecular weight compound such as EMB might provide a new avenue to impair NS pro-metastatic activity in the context of a brain oncologic disease.

Although further work is needed to understand the details of the interaction between EMB and the NS conformers, our results show for the first time that a small molecule is able to dissolve already formed NS polymers, and to inhibit the formation of long-chain polymers, shedding novel suggestions for the search drug leads against FENIB. Furthermore, inhibition of NS by small molecules may also find interesting applications in distinct human pathologies. Although EMB has been ascribed many biological activities, ranging from modulation of apoptosis to modification of gene expression^38^,^39^,^40^, which could discourage its direct use as a drug, our work provides an original proof-of-principle for future applications of small molecules interventions for FENIB and other serpinopathies.

## Methods

### *NS expression and purification.*

NS was expressed and purified according to^22^.

### Production of NS conformers

Lat and Pol were prepared by incubating Nat overnight (0.5 and 4 mg/ml, respectively) at 45 °C in 10 mM Tris, 50 mM KCl, pH 8.0. The two species were subsequently purified by SEC (Superdex 200 10/300 GL, GE Healthcare, Little Chalfont, UK). The Cle conformer was prepared as described in^22^.

### Incubation with Embelin

In order to prepare EMB solutions with a controlled concentration we tested two strategies. In the first approach, following a conventional route, EMB was solved into a high concentration stock solution in dimethyl-sulfoxide (DMSO), up to EMB solubility in DMSO (120 mM) and then diluted 1:1000 in the final NS solution. Such a high dilution is required since a DMSO content higher than 0.5% (v/v) affects NS secondary structure and at any concentration DMSO has a pro-polymerisation effect. Moreover, the actual EMB solubility in 0.1% DMSO solution is even lower than 120 μM thus not playing any beneficial effects on EMB solubility. The above technical issues do not allow reaching a high stoichiometric ration between EMB and NS, which is necessary to efficiently drive their interaction.

In order to overcome these experimental constraints, we envisaged an alternative strategy able to avoid the presence of organic solvents and enhance the interaction of EMB with NS. EMB powder (Sigma) was directly added to the NS solution (10 mM Tris HCl, 50 mM KCl, pH 8.0) and thoroughly dissolved until reaching saturation conditions. The solution was then immediately centrifuged at 4 °C for 10 minutes at 20000 rcf in order to remove the excess of insoluble EMB. Due to the limited EMB solubility such method allowed to reproducibly reach the saturated concentration of 1.5 mM. EMB concentration was monitored by measuring EMB absorbance at 325 nm (ε = 24000 M^−1^ cm^−1^ at pH 7.4). The supernatant was then used for the experiments described below. Specific incubations of Nat were performed at different temperatures as described in the figure legends: overnight at 4 mg/ml, or at 1.7 mg/ml. In all cases, at the end of the incubation, the NS/EMB samples were centrifuged at 4 °C for 10 minutes at 20000 rcf and the supernatant analysed by different techniques.

### SEC-FPLC

All the analytical SEC runs were performed using a Superdex200 10/300 GL column (GE Healthcare) in 10 mM Tris HCl, 50 mM KCl, pH 8.0 buffer.

### SEC-HPLC

NS samples consisting of 200 μL at 4 mg/mL were separated using an HPLC system composed by a Waters 515 HPLC Pump connected with different detectors, as needed.

### SEC-MALS

Molar mass of low molecular weight species generated after incubation of Nat with EMB were characterized by connecting on-line the SEC-HPLC system with a Dawn® Heleos® Multi Angle Light Scattering (Wyatt, Santa Barbara, CA, USA), a Waters 2487 Dual λ Absorbance Detector and a Optilab® T-rEX Refractive Index Detector (Wyatt, Santa Barbara, CA, USA). Molar mass at different volumes of elution was calculated by Astra software (v. 5.3.4.18, Wyatt, Santa Barbara, CA, USA) by using 0.185 as dn/dc value.

### Multi-wavelength HPLC

Multi-wavelength HPLC runs were carried out by connecting on-line the SEC-HPLC system with a Waters 996 Photodiode Array Detector. Chromatograms at specific wavelengths (280 and 340 nm for proteins and EMB, respectively) and UV-vis spectra of peaks were extrapolated by means of Waters Empower Pro software. NS does not display any absorbance at 340 nm and EMB incubated in the absence of NS is eluted at much higher volume in SEC, as typical for small organic molecules (data not shown).

### *Native polyacrylamide gel electrophoresis.*

Native-PAGE gels, consisting of a 7.5% acrylamide resolving gel and a 5% stacking gel, were run at 90V on ice to prevent sample denaturation and polymer dissociation in standard sample and running buffers according to^41^. Nat concentration was 4 mg/ml in both samples.

### *ESI-MS analysis*.

Samples for ESI-MS analyses contained 100 mM ammonium acetate pH 8, and either native or pre-incubated NS at 10–20 μM total protein concentration. The analyses were performed on a hybrid quadrupole time-of-flight mass spectrometer (QSTAR Elite, AB-Sciex, FosterCity, CA, USA), equipped with a nano-ESI source. Samples were infused through metal-coated borosilicate capillaries, with emitter tips of 1 μm internal diameter (Proxeon, Odense, Denmark). The following instrumental setting was applied: positive ion-mode, declustering potential 300 V, ion spray voltage 1.3 kV and curtain-gas pressure 20 psi. Unusually high values of declustering potential were necessary to counteract adduct formation during the electrospray. The sample source and the instrument interface were kept at room temperature. Data were analyzed using the Analyst 2.0 software.

### *Photoluminescence*.

PL measurements were performed by incubating 5 μM NS samples (with or without EMB incubation) on a Jasco FP-6500 spectrofluorimeter using a quartz cuvette with a 1.5 mm optical path. The temperature was controlled within 0.05 °C with a temperature-controlled recirculating bath. The normalised emission bands PL(λ) were measured with a 275 nm excitation wavelength, buffer subtracted and normalised by the total PL area. The first momentum λ_1_ of the emission band was calculated as λ_1_** = **∫ λ PL(λ)dλ.

### *Circular Dichroism*.

CD experiments were carried out on a J-810 spectropolarimeter (JASCO Corp., Tokyo, Japan) equipped with a Peltier system for temperature control using a 0.1 cm path length cuvette. The CD spectra shown in figure 5A have been measured on 12.3 μM solution of Nat and Pol NS in 50 mM KCl, 10 mM Tris-HCl, pH 8.0, with or without incubation with EMB. NS samples were incubated in saturated EMB solution as described above and then washed by spin columns to remove excess EMB before collecting the CD spectrum. The Pol NS samples (with or without EMB) were formed upon incubation of Nat solution at 55 °C for two hours, residual monomeric NS was removed by spin columns (cut-off 100kDa). All measurements were performed at pH 8.0, 20 °C, with an optical path of 0.1 cm.

For the temperature ramp experiments, ellipticity at 218 nm wavelength was recorded from 20 to 95 °C (temperature slope 1.0 °C/min) on NS samples (Nat, Lat and Cle with or without EMB) in 50 mM KCl, 10 mM Tris-HCl, pH8.0, at a NS concentration of 0.25 mg/ml. NS samples were incubated for 30 minutes at 4 °C in a saturated EMB solution, then centrifuged for 10 minutes at 20000 rcf before starting the temperature ramps.

### *Sandwich ELISA.*

The affinity of anti-NS monoclonal antibodies (mAb)^19^ for NS conformers with and without EMB was quantified by sandwich ELISA as described previously^19^. Briefly, 96-well plates (Corning Inc., Costar 3590) were coated with antigen-purified rabbit polyclonal anti-NS antibody (2 μg/ml), washed (0.9% w/v NaCl, 0.05% v/v Tween20) and blocked with blocking buffer (PBS, 0.25% w/v bovine serum albumin, 0.05% v/v Tween20, 0.025% w/v Na azide). Gel filtered NS conformers and NS/EMB complexes were diluted in blocking buffer and incubated for 2 h. After washing, wells were incubated with each mAb (1A10, 10B8, 10G12 and 7C6) from 10 μg/ml with serial dilutions. Rabbit anti-mouse IgG-HRP labeled antibody was used for detection with TMB substrate solution, and HRP activity was measured in a GloMax plate reader (Promega) at 450 nm. The interplate and intraplate coefficient of variation were both less than 5%.

### *NS inhibition kinetics.*

The progress curves for the inhibitory reaction of NS on 2ctPA (American Diagnostica) were determined in the presence of the chromogenic substrate H-D-Ile-Pro-Arg-p-nitroanilide (IPR-pNA; Chromogenix) by recording the formation of pNA upon cleavage of the substrate. Experiments were performed at 25 °C in 50 mM Tris, 10 mM Na_2_HPO_4_, 150 mM NaCl, and 0.1% Tween (pH 7.4). Buffer, NS (60 nM), and substrate (250 μM) were mixed in a 2 ml cuvette and reactions were initiated by addition of a fixed amount of 2ctPA (1 nM). Product accumulation was continuously recorded by a Cary 4E spectrophotometer (Varian, Inc.) at 405 nm. The experiment, as shown in Fig. 6, was repeated three times.

### *Formation of NS/tPA acyl–enzyme complex.*

Samples of different NS species were incubated with 2ctPA at a 6:1 (NS:2ctPA) molar ratio in the same buffer as for the chromogenic assays. The reactions were stopped at time intervals by addition of a denaturing buffer containing Na dodecyl-sulphate and β-mercaptoethanol followed by heating for 10 min at 100 °C. Samples were analysed by SDS-PAGE in 10% separating polyacrylamide gels. After electrophoresis, proteins were stained with SYPRO Ruby (Molecular Probes) and visualized with a Typhoon 9200 laser scanner. Protein quantification was performed using ImageQUANT software (GE Healthcare Life Sciences).

## Additional Information

**How to cite this article**: Saga, G. *et al.* Embelin binds to human neuroserpin and impairs its polymerisation. *Sci. Rep.* **6**, 18769; doi: 10.1038/srep18769 (2016).

## Supplementary Material

Supplementary Information
