# Supplementary material for: Embelin binds to human neuroserpin and impairs its polymerisation
Source: Sci Rep. 2016 Jan 6;6:18769. doi: 10.1038/srep18769 (PMC4702122; doi:10.1038/srep18769)
Supplement: Supplementary Information [file srep18769-s1.pdf]

*Supplementary Material*

# Embelin binds to human neuroserpin and impairs its pathologic polymerisation

Giorgia Saga, Fabio Sessa, Alberto Barbiroli, Carlo Santambrogio, Rosaria Russo, Michela Sala, Samuele Raccosta, Vincenzo Martorana, Sonia Caccia, Rosina Noto, Claudia Moriconi, Elena Miranda, Rita Grandori, Mauro Manno, Martino Bolognesi, Stefano Ricagno

**A**

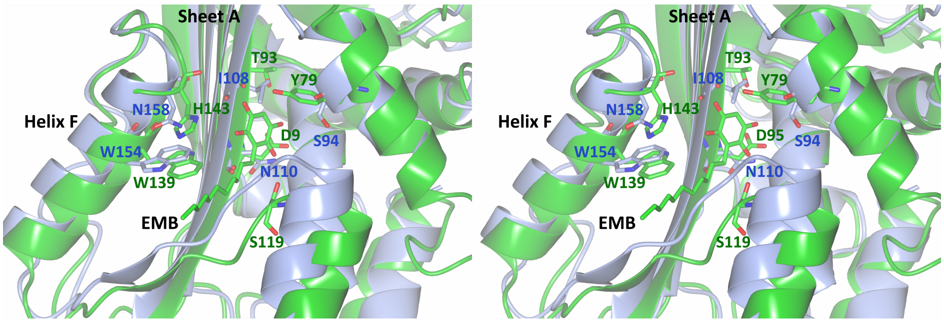


**B**

```
NS                  PTGATFPEEAIADLSVNMYNRLRATGEDENILFSPLSIALAMGMMELGAQ
PAI1                PSY---VAHLASDFGVRVFQQVAQASKDRNVVFSPYGVASVLAMLQLTTG
                    *:      .  :*:.*.:::::  :.:*.*::*** .:* .:.*::* :

NS                  GSTQKEIRHSMGYDSLKNGEEFSFLKEFSNMVTAKESQYVMKIANSLFVQ
PAI1                GETQQQIQAAMGFKIDDKGMAPALRHLYK-ELMGPWNKDEISTTDAIFVQ
                    *.**::*: :**:.  .:*   :: : :.  : .  .:  :. ::::***

NS                  NGFHVNEEFLQMMKKYFNAAVNHVDFSQNVAVANYINKWVENNTNNLVKD
PAI1                RDLKLVQGFMPHFFRLFRSTVKQVDFSEVERARFIINDWVKTHTKGMISH
                    ..::: : *:  : : *.::*::****:   .   **.**:.:*:.::..

NS                  LVSPRDFDAATYLALINAVYFKGNWKSQFRPENTRTFSFTKDDESEVQIP
PAI1                LLGTGAVDQLTRLVLVNALYFNGQWKTPFPDSSTHRRLFHKSDGSTVSVP
                    *:..  .*  * *.*:**:**:*:**: *  ..*:   * *.* * *.:*

NS                  MMYQQGEFYYGEFSDGSNEAGGIYQVLEIPYEGDEISMMLVL-SRQEVPL
PAI1                MMAQTNKFNYTEFTT---PDGHYYDILELPYHGDTLSMFIAAPYEKEVPL
                    ** * .:* * **:      *  *::**:**.** :**::.   .:****

NS                  ATLEPLVKAQLVEEWANSVKKQKVEVYLPRFTVEQEIDLKDVLKALGITE
PAI1                SALTNILSAQLISHWKGNMTRLPRLLVLPKFSLETEVDLRKPLENLGMTD
                    ::*  ::.***:..* ..:.:    : **:*::* *:**:. *: **:*:

NS                  IFIK-DANLTGLSDNKEIFLSKAIHKSFLEVNEEGSEAAAVSGMIAISRM
PAI1                MFRQFQADFTSLSDQEPLHVALALQKVKIEVNESGTVASSSTAVIVSARM
                    :* : :*::*.***:: :.:: *::*  :****.*: *:: :.:*. :**

NS                  AVLYPQVIVDHPFFFLIRNRRTGTILFMGRVMHPETMNTSGHDFEEL
PAI1                AP--EEIIIDRPFLFVVRHNPTGTVLFMGQVMEP-------------
                    *    ::*:*:**:*::*:. ***:****:**.*
```

**Figure S1**: **(A)** Superposition (stereo view) of EMB binding site in the PAI-1/EMB complex (PDB code 3UT3, in green) with the structure of native NS (PDB code 3F5N, light blue). PAI-1 residues interacting with EMB and the corresponding NS residues are shown as sticks. **(B)** Sequence alignment of NS and PAI-1, the sequence identity is 33%.

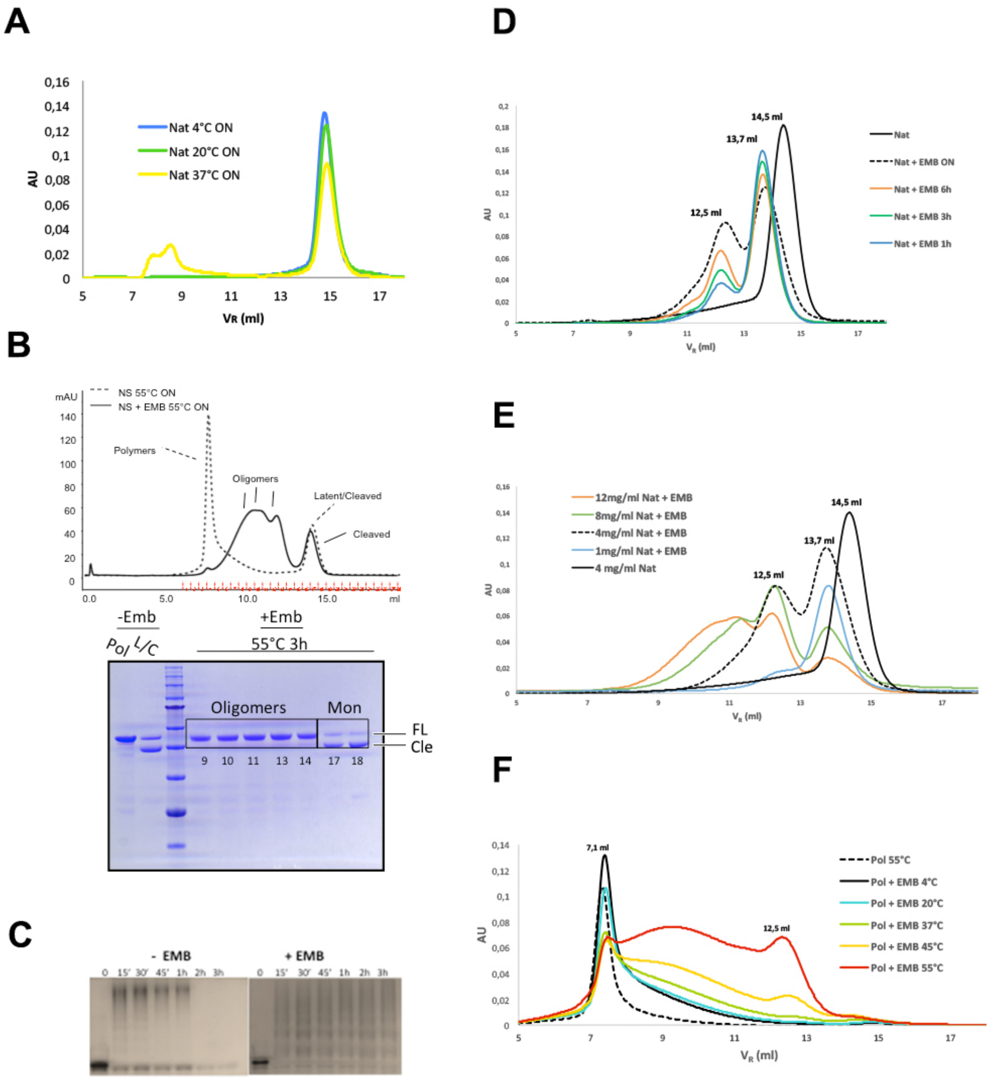


**Figure S2**: **(A)** SEC profile (Superdex 200 GL 10/300) of Nat incubated overnight at three different temperatures in absence of EMB. **(B)** Above, SEC profile after incubation of Nat at 55 °C overnight with and without EMB (continuous and dashed curves, respectively). Below, SDS-PAGE analysis of SEC fractions from the two incubations. The SDS-PAGE analysis of the two peaks eluted after incubation without EMB reveals that the monomeric peak is a mixture of uncleaved (Lat) and of Cle. The SDS-PAGE samples of the incubation with EMB provides several data: (i) there is no protein degradation during incubation in presence of EMB; (ii) NS oligomers formed are purely constituted by uncleaved NS; (iii) the presence of EMB inhibits the formation of Lat: the monomeric fraction at the end of the incubation consists almost entirely of Cle (FL and Cle labels on the right of the SDS-PAGE stand for full length and cleaved). This incubation was performed using a NS solution containing about 70% of Nat and 30% of Cle in order to highlight the different behaviours of Nat and Cle in presence or absence of EMB. **(C)** Native PAGE of Nat incubated at 55 °C alone (-EMB) and with EMB (+EMB) monitored during 3 hours. **(D)** SEC profiles (Superdex 200 GL 10/300) of Nat (4 mg/ml) incubated with EMB at 20 °C analysed at the indicated times. **(E)** SEC

profiles (Superdex 200 GL 10/300) of Nat solutions at different concentrations (1-12 mg/ml) incubated with EMB at 20 °C overnight. **(F)** Effect of EMB on Pol (1.7 mg/ml) incubated at different temperatures overnight (4, 20, 37, 45, 55 °C) monitored by SEC. As negative control the SEC profile of Pol incubated at 55 °C overnight without EMB is shown, indicating that prolonged incubation at high temperature has no effect on Pol stability.

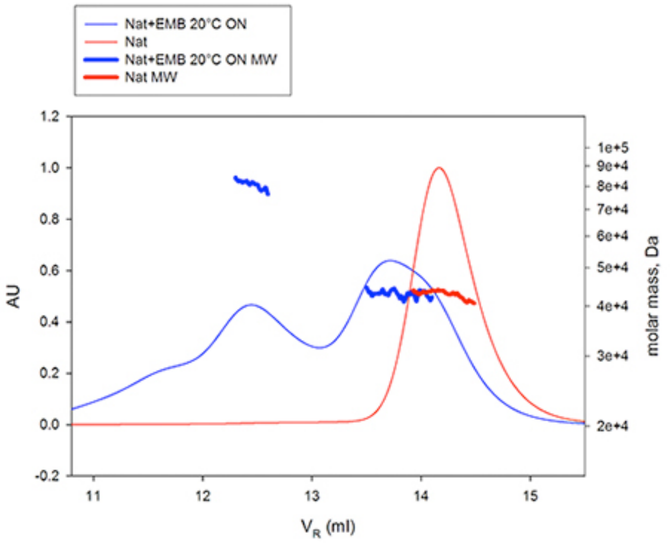


**Figure S3:** SEC profile of Nat (red line) and Nat incubated overnight at 20 °C with EMB (blue line). The thick red and blue lines indicate the molecular weight estimated by MALS of Nat and of the two major peaks ($V_R$ =13.7 and 12.5 ml) after incubation, respectively.

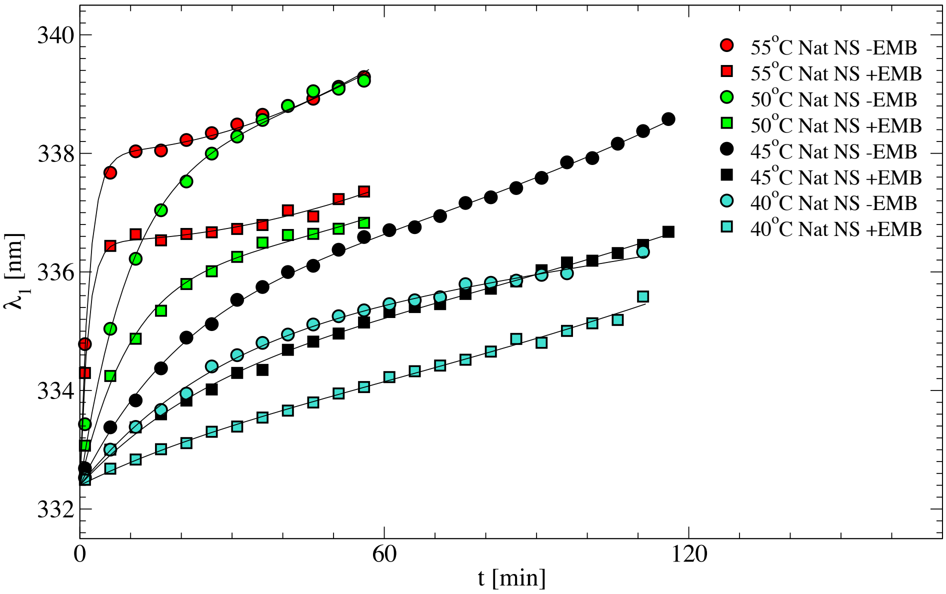


**Figure S4:** Kinetics of NS polymerization monitored by PL emission (excitation wavelength 275 nm). First moment of the emission spectra of NS (7.5 μM, 10 mM Na phosphate pH 7.4, 100mM NaCl) incubated as indicated. Nat samples were incubated at high temperature either alone (circles) or in presence of EMB in a molar excess of 1:5 NS:EMB (squares).

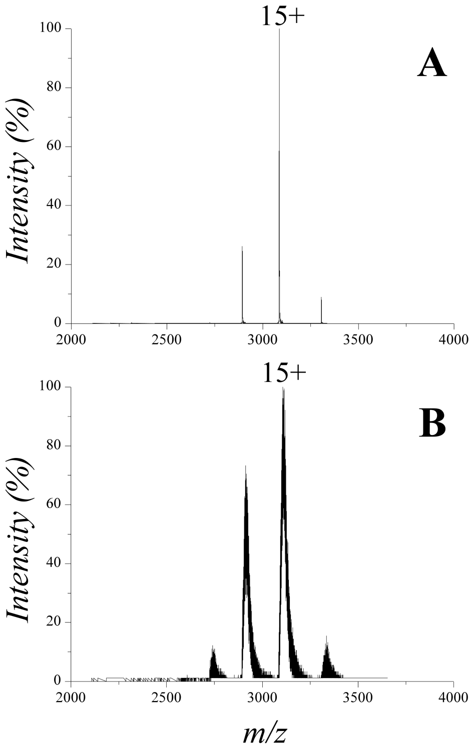


**Figure S5:** ESI-MS spectra of neuroserpin 20 μM in ammonium acetate 100 mM pH 8 after incubation of 1h at 20 °C, in the absence (A) and presence (B) of embelin at saturating concentration. The measured molecular weight is 46285.2 Da (A) and 46576.0 Da (B). The most intense peak in each panel is labeled by the corresponding charge state.

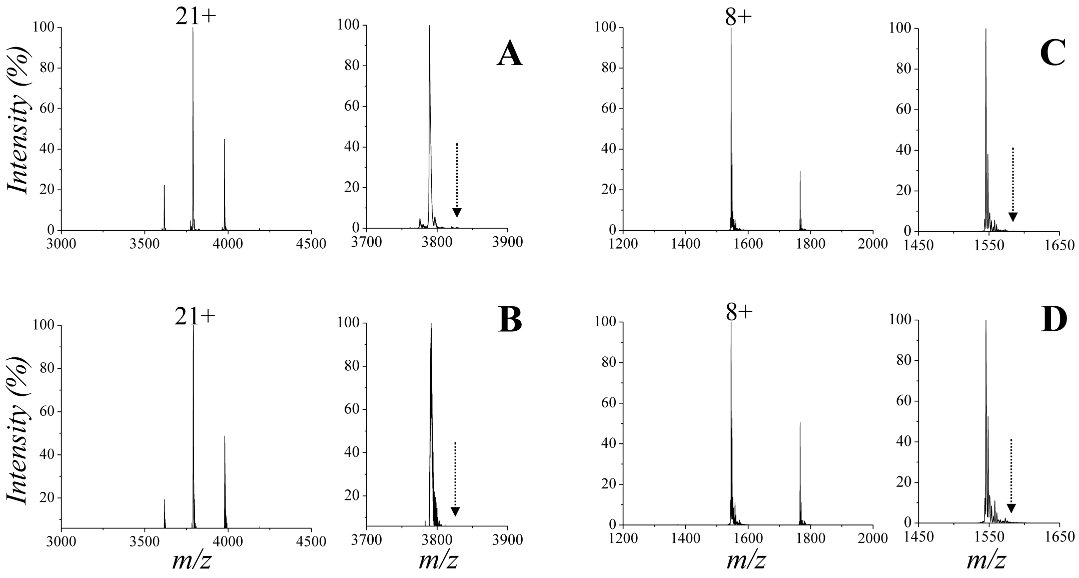


**Figure S6:** ESI-MS spectra of 20 µM transferrin (A, B) and cytochrome C (C, D) in 100 mM ammonium acetate pH 8, after 1h incubation at 20 °C in the absence (A, C) or presence (B, D) of EMB at saturating concentration and subsequent desalting by spin columns. The most intense peak in each panel is labelled by the corresponding charge state and magnified in the adjacent panel, where a dotted arrow indicates the expected position of the peak corresponding to the 1:1 protein:EMB complex. The measured molecular weight after incubation does not indicate any complex formation between transferrin/cytochrome C and EMB.

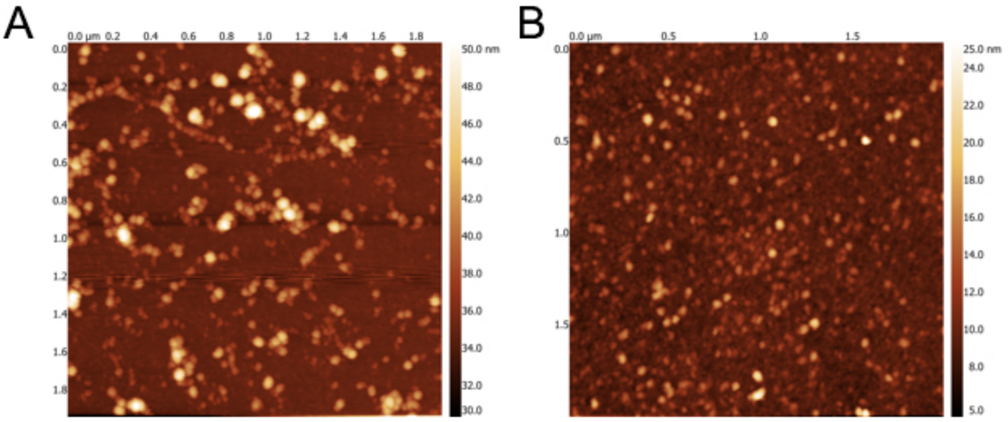


**Figure S7:** Atomic Force Microscopy (AFM). (A) NS Pol. (B) NS/EMB oligomers. Pol were formed by incubating 10uM NS samples at 55 °C for 3 hours, while oligomers were obtained by incubating 70 μM NS samples at 45 °C overnight with EMB. All the samples were transferred in Millipore Super-Q water, diluted to a final concentration of 400 nM or 100 nM for polymers and oligomers, respectively. A 30 µL drop of each sample was deposited on a mica substrate and dried by a gentle nitrogen flux to remove most of the surface water. Images of protein aggregates were recorded by a JPK Nanowizard 3, operating in air tapping mode (resolution 512 × 512, scan rate 0.5 Hz). We used rigid cantilevers (resonance frequency 320 kHz), equipped with silicon tips with a nominal radius of curvature of 8 nm.
